# Beyond the Bench: Virtual School

**Published:** 2005-10

**Authors:** Tanya Tillett

In today’s world of high-speed interconnection, technology in the classroom helps keep students interested and engaged in the learning process. Taking advantage of this favorable avenue of instructional opportunity, the Community Education and Outreach Program (COEP) of the NIEHS Center in Molecular Toxicology at Vanderbilt University, in conjunction with the university’s Center for Science Outreach (CSO), has developed an innovative interactive videoconference teaching program known as “Virtual Scientist in the Classroom.” The program creates a direct connection between Vanderbilt University faculty and students all over the country, allowing university researchers to lecture on environmental health topics related to the work they are performing in their own laboratories.

“Through the center’s involvement with outreach and education, we are able to provide reliable, up-to-date, and cutting-edge science to classrooms throughout Tennessee and the U.S.,” says Bradley Hawkins, the COEP director. “In addition, the students are able to interact with our researchers in a manner that was not available just a few years ago.”

The program relies on volunteer faculty with diverse research interests—neuroscience, diabetes mellitus, biomedical engineering, physics, molecular toxicology, and chemistry, for example—who create their own presentations and conduct the sessions in real time in the CSO virtual learning studio (all presentations are taped and archived for future multi-classroom sessions). The topics for presentations to date have included how medicines are developed, how chemicals damage DNA, and the importance of micronutrients. The format of each session is left to the discretion of the expert presenters, and may include anything from PowerPoint slides to movie clips, live virtual tours of lab facilities, even real-time electrocardiograms. One physics professor presented the theory of relativity in character as Albert Einstein.

The sessions of 30–45 minutes can be presented to one school at a time or to multiple site audiences. Scientist–student interaction is a main component of the sessions; questions and feedback from students are expected and encouraged. By using a communications bridge capable of connecting to multiple sites within a videoconference session, the researchers open up the world of scientific discovery to students in classrooms all over the state of Tennessee and beyond. When the program was created in 1999, it primarily reached out to middle and high school students in Tennessee, but has grown to include videoconferences to children in 75 schools in 20 states.

Typically four to six sessions on varied topics are offered each semester. Teachers can find complete descriptions of each session online at http://www.vanderbilt.edu/cso/ and can register there for each session. The sessions are free for Tennessee students, although a charge is applied for out-of-state schools. Once teachers have registered, they can download supplemental lesson material and will receive e-mailed confirmation and detailed instructions for participation. In a new feature, researchers answer questions that arise after each session, and the 1- to 2-minute video response also is archived on the site.

The continued commitment and enthusiasm of the contributing faculty members is a cornerstone of the program, and helps keep the videoconference sessions relevant and timely. “I believe that as researchers we need to take an active role in helping to educate and inform the public about issues related to adverse health outcomes upon exposure to poisons, to educate the public about sources of poisons in food and air, and the mechanisms by which they affect our health,” says Michael Aschner, a professor of pediatrics and pharmacology who has presented on the subject of chemical insults to the brain. Hopefully, he says, educational outreach programs can help bridge the gap between public understanding and public perception of toxicology.

## Figures and Tables

**Figure:**
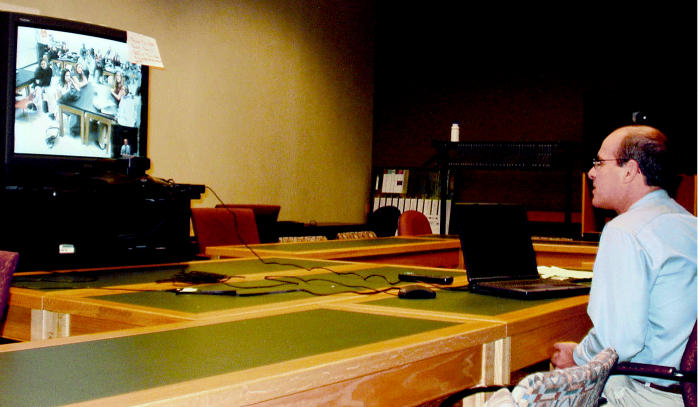
M(aven)TV? Vanderbilt University specialists use the Internet to connect kids with science straight from the lab.

